# Efficacy and tolerability of adjunctive lacosamide in patients aged <4 years with focal seizures

**DOI:** 10.1002/acn3.52004

**Published:** 2024-02-20

**Authors:** Iryna Makedonska, Yu‐Tze Ng, Cynthia Beller, Ali Bozorg, János Csikós, Carrie McClung, Holger Moeltgen, Mark Kristof Farkas, the SP0967 Study Group, Conceição Campanário da Silva Pereira Almeida, Anna Altmann, Elena Arefieva, Susan Arnold, Mihaela Axente, Maria Giuseppina Baglietto, Sophio Bakhtadze, Domenica Immacolata Battaglia, Liudmila Belova, Marianne Berényi, Cornelia Calcii, Dezhi Cao, Juan Fernando Capristo Gonzales, Hugo Ceja Moreno, Yuriy Chomolyak, Jo Janette De la Calzada, Anne de Saint Martin, Dmytro Delva, Tayard Desudchit, Gabriella Di Rosa, Argirios Dinopoulos, Milda Endziniene, Viktor Farkas, Jose Ferreira, José Carlos Ferreira, András Fogarasi, Cassiano Mateus Forcelini, Hadassa Goldberg‐Stern, Tiziana Granata, Ioana Grigore, Christian Paul Guzman Astorga, Jose Antonio Infante Cantu, Li Jiang, Yuwu Jiang, Pongkiat Kankirawatana, Yulia Karakulova, Olga Khaletskaya, Volodymyr Kharytonov, Heung Dong Kim, Ki Joong Kim, Marija Knezevic Pogancev, Liudmila Kraeva, Ruzica Kravljanac, David Kvernadze, Alla Kyrychenko, Volodymyr Kyrychenko, Wang‐Tso Lee, Jianmin Liang, Elmer Guillermo López‐Meza, Marissa Lukban, Olga Lvova, Maša Malenica, Maria Margherita Mancardi, Volodymyr Martyniuk, Gia Melikishvili, Richard Morse, Sylvia Napuri, Dimitrije Nikolic, Vilem Novak, Liliana Maria Nussbaum, Claudio Palacios, Pavel Pilipenko, Barbara Prawdzic‐Senkowska, Igor Prpic, Roshan Raja, Olga Shestakova, Maria Strachunskaya, Roberto Alfonso Suástegui Román, Nino Tatishvili, Salvador Vázquez Fuentes, Federico Vigevano, Gabriela Adriana Visa, Yi Wang, Elza Márcia Yacubian, Jianmin Zhong

**Affiliations:** ^1^ Municipal Non‐profit Enterprise City Children's Clinical Hospital #6 of Dnipro City Council Dnipro Ukraine; ^2^ Baylor College of Medicine/The Children's Hospital of San Antonio San Antonio Texas USA; ^3^ UCB Pharma Morrisville North Carolina USA; ^4^ UCB Pharma Monheim am Rhein Germany; ^5^ Pediatric Center, MTA Center of Excellence Semmelweis University Budapest Hungary; ^6^ Present address: University of Missouri School of Medicine Columbia Missouri USA; ^7^ Present address: Otsuka Pharmaceutical Development & Commercialization, Inc. Rockville Maryland USA

## Abstract

**Objective:**

Primary objective was to evaluate efficacy of lacosamide administered concomitantly with 1–3 antiseizure medications in young children with uncontrolled focal (partial‐onset) seizures.

**Methods:**

Double‐blind, parallel‐group trial (SP0967: NCT02477839/2013‐000717‐20) conducted between June 2015 and May 2020 at hospitals and clinics in 25 countries. Patients (aged ≥1 month to <4 years) with uncontrolled focal seizures were randomized 1:1 to adjunctive lacosamide or placebo using an interactive voice/web response system and stratified by age. After a 20‐day titration period, patients who reached target‐dose range (8–12 mg/kg/day) entered a 7‐day maintenance period. Region‐specific primary efficacy variables were based on ≤72‐h video‐electroencephalograms: change in average daily frequency (ADF) of electrographic focal seizures as measured on end‐of‐maintenance video‐electroencephalogram versus end‐of‐baseline video‐electroencephalogram (United States); 50% responder rate (≥50% reduction in ADF of focal seizures) during maintenance (European Union).

**Results:**

In total, 255 patients were randomized (lacosamide/placebo: 128/127) and received ≥1 trial medication dose. Percentage reduction in ADF of focal seizures for lacosamide (116 patients) versus placebo (120 patients) was 3.2% (95% confidence interval = −13.6 to 17.5, *p* = 0.69). 50% responder rate was 41.4% for lacosamide (116 patients), 37.5% for placebo (120 patients) (*p* = 0.58). Treatment‐emergent adverse events were reported by 44.5% of lacosamide‐treated patients (placebo 51.2%).

**Interpretation:**

Adjunctive lacosamide did not show superior efficacy versus placebo in young children with focal seizures. However, efficacy variables were potentially affected by high variability and low reliability between readers in video‐electroencephalogram interpretation. Lacosamide was generally well tolerated; safety profile was acceptable and consistent with that in adults and children aged ≥4 years.

## Introduction

Epilepsy is the most common neurological condition in childhood.^1^ Approximately 1 out of 150 children is diagnosed with epilepsy during the first 10 years of life, and the incidence is highest during infancy.^1^ Efficacious and safe antiseizure treatment is particularly important for children because refractory epilepsies and a high seizure burden during brain development are associated with severe cognitive, behavioral, and motor delay.^2^ However, 25% to 30% of children with epilepsy have medically refractory epilepsy despite new antiseizure medications (ASMs) being available.^3^

Lacosamide (LCM; Vimpat^®^, UCB Pharma, Brussels, Belgium) is indicated as adjunctive therapy and monotherapy for focal (partial‐onset) seizures in patients aged 1 month and older in the United States (US),^4^ and in patients aged 2 years and older in the European Union (EU).^5^ LCM is also approved as adjunctive therapy for primary generalized tonic–clonic seizures in patients aged ≥4 years in the US and EU.^4^, ^5^ At the time that this Phase III, double‐blind, randomized, placebo (PBO)‐controlled trial (SP0967: ClinicalTrials.gov identifier NCT02477839, EudraCT number 2013‐000717‐20) was conducted, LCM was not approved for use in patients aged <4 years. The primary objective of SP0967 was to evaluate the efficacy of LCM administered concomitantly with 1–3 ASMs in children aged ≥1 month to <4 years with uncontrolled focal seizures. Evaluation of safety and tolerability was a secondary objective. The lessons learned regarding the use of video‐EEG (video‐electroencephalogram) as an efficacy measure are discussed in a companion paper.^6^

## Methods

### Standard protocol approvals, registrations, and patient consents

The trial was conducted in accordance with applicable regulatory and International Council for Harmonisation‐Good Clinical Practice (ICH‐GCP) requirements, the Declaration of Helsinki, and local laws. The protocol and amendments were reviewed by a national, regional, or independent ethics committee or institutional review board. Written informed consent was provided by parents or legal representatives of all patients. The trial was registered with ClinicalTrials.gov (NCT02477839) and EudraCT (2013‐000717‐20).

### Patients

SP0967 was a Phase III, multicenter, double‐blind, randomized, PBO‐controlled, parallel‐group trial conducted at hospitals and clinics in 25 countries across Asia, Europe, Middle East, North America, and South America between June 5, 2015 and May 28, 2020. The protocol and statistical analysis plan are available in the study's ClinicalTrials.gov record. In total, 89 sites screened patients and 75 sites enrolled/treated patients in the safety set (SS), which included all randomized patients who took ≥1 dose of trial medication.

Patients were eligible for enrollment if they were aged ≥1 month (i.e., 4 weeks after full term [defined as 37 weeks gestational age]) to <4 years. For preterm infants aged <1 year, the corrected gestational age was used to determine eligibility. Additional inclusion criteria included a diagnosis of epilepsy with focal seizures, with ≥1 prior EEG and ≥1 magnetic resonance imaging/computerized tomography scan consistent with this diagnosis; body weight of ≥4 to <30 kg at visit 1; ≥2 focal seizures with or without secondary generalization during each consecutive 7‐day period during the 2 weeks before visit 1; ≥2 focal seizures with or without secondary generalization during the end‐of‐baseline period (EOB) video‐EEG (see Outcome Variables for details); and a stable (concurrently or sequentially) dosage regimen of 1–3 ASMs, with the dosage regimen of concomitant ASM treatment kept constant for ≥2 weeks before visit 1 (a stable daily dosage regimen of a concomitant benzodiazepine was considered a concomitant ASM). Vagus nerve stimulators (VNSs) were allowed and not counted as a concomitant ASM. The VNS must have been implanted for ≥6 months before visit 1, and device settings must have been kept stable for ≥2 weeks before visit 1 and during the baseline, treatment, and transition periods. Use of the VNS magnet was permitted.

Exclusion criteria included participation in another study of an investigational medicinal product or device within the previous 2 months; previous LCM treatment that was stopped because of a lack of efficacy or an adverse event (AE); febrile seizures only; Lennox–Gastaut syndrome, epilepsia partialis continua, primary generalized epilepsy, Dravet syndrome, or seizures not of focal origin; or history of generalized convulsive status epilepticus within the previous 2 months. Additional exclusion criteria, withdrawal criteria, and important protocol amendments are described in eMethods in Supplementary Materials.

### Treatment schedule

**Figure 1:**
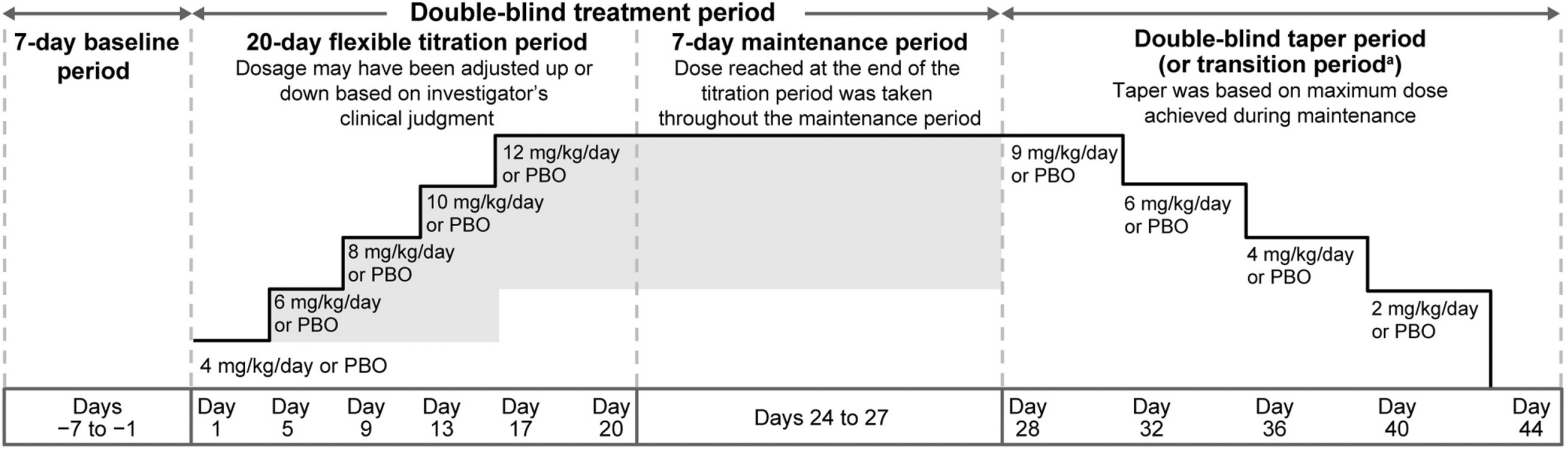
SP0967 trial design. PBO, placebo. ^a^A blinded 12‐day transition period was required for eligible patients who completed the maintenance period and were entering the open‐label extension trial EP0034. During this period, patients on lacosamide remained on their maintenance dose, whereas patients on PBO were transitioned in a double‐blind manner to lacosamide 8 mg/kg/day. At visit 1 of EP0034 (the final transition visit of SP0967), all patients were to be transitioned to lacosamide 10 mg/kg/day.

The trial comprised a 7‐day baseline period (starting with the screening visit [visit 1]), 20‐day blinded titration period, and 7‐day blinded maintenance period (Fig. 1). Patients who completed the maintenance period were eligible to enroll in an open‐label extension trial (EP0034: NCT01964560, 2012–005012‐26). Patients entering the extension trial had a 12‐day blinded transition period; patients who did not enroll in the extension trial had a blinded taper period followed by a 30‐day safety follow‐up period.

At visit 1 (Day −7), patients meeting the selection criteria began the baseline period, at which time a video‐EEG was performed in an inpatient setting. After completion of the EOB video‐EEG at visit 3 (Day 1), which was assessed for seizure count by the investigator, sub‐investigator, or qualified designated reader at the site, eligible patients were randomized 1:1 to LCM or PBO and stratified by age category (≥1 to <6 months, ≥6 months to <1 year, ≥1 to <2 years, ≥2 to <4 years). Details of randomization are given in eMethods.

Trial medication was administered twice daily, at approximately 12‐h intervals. During titration to target dose (8–12 mg/kg/day), doses started at 4 mg/kg/day and were then increased by 2 mg/kg/day every 4 days. Dosage could be adjusted up or down (to a minimum of 4 mg/kg/day) based on tolerability, with no limit to the number of titration steps or dose holds. Patients who did not reach the minimum target dose (8 mg/kg/day) for the final 3 days of the titration period were withdrawn. No dose adjustments were allowed during the maintenance period.

### Outcome variables

Region‐specific primary and secondary efficacy variables were based on video‐EEGs (≤72 h of continuous recording, with every attempt to obtain ≥48 h of interpretable recording), which were evaluated for seizure counts locally by the investigator, sub‐investigator, or qualified designated reader at the site. Per the original protocol, a central reader was responsible for reviewing all video‐EEGs to obtain the seizure counts for primary and secondary efficacy analyses, and the investigators were responsible for determining patient eligibility in real time based on the requirement for ≥2 focal seizures on the EOB video‐EEG. However, a review of the first group of 7 EOB video‐EEGs assessed by the central reader showed a high degree of discordance in seizure counts between the central reader and investigators: for 71% of the patients that the investigators had considered to have ≥2 focal seizures on the EOB video‐EEG, the central reader did not count ≥2 focal seizures.^6^ Therefore, partway through the trial, the protocol was amended to remove the central reader, and to assign the responsibility for determining seizure counts for the efficacy analyses (including seizure counts for patients who had already completed the trial) to the investigators.

Per protocol, electrographic seizures were defined as recognizable ictal patterns on an EEG involving ≥2 contiguous electrodes; the seizures were initiated as a unilateral or strongly asymmetric abnormal epileptiform discharge lasting a total of >10 s. Focal seizure frequency was based on electrographic seizures for infants aged ≥1 to ≤6 months, and on electrographic seizures with an accompanying clinical correlate for children aged >6 months to <4 years.

For the US, the primary efficacy variable was contingent on the percentage of patients who discontinued from the trial after the first dose of trial medication but before performance of the end‐of‐maintenance period (EOM) video‐EEG (i.e., early discontinuation). Because ≤10% of patients discontinued early from the trial, the primary efficacy variable for the US was change in average daily frequency (ADF) of electrographic focal seizures as measured on the EOM video‐EEG versus the EOB video‐EEG. For the EU, the primary efficacy variable was the 50% responder rate (the proportion of patients with a ≥50% reduction in ADF of electrographic focal seizures) during the maintenance period (this was also the contingent primary efficacy variable for the US).

Secondary efficacy variables were the percentage and absolute change in ADF of electrographic focal seizures from the EOB video‐EEG to the EOM video‐EEG; the proportion of patients who achieved “seizure‐free” status (from all seizure types, and from focal seizure types only); and the proportions of patients with a ≥25% to <50%, 50% to 75%, or >75% reduction, with no change (<25% reduction to <25% increase), or with a ≥25% increase in ADF of electrographic focal seizures from the EOB video‐EEG to the EOM video‐EEG. Other efficacy variables were Clinical Global Impression of Change (GIC), Caregiver's GIC, and change from baseline in Pediatric Quality of Life Inventory (PedsQL) health summary score at the EOM.

Primary safety variables included AEs reported spontaneously by the patient's parent(s) and/or legal representative(s)/caregiver(s) (in accordance with local regulation) or observed by the investigator, and patient withdrawals because of AEs. AEs were coded using the Medical Dictionary for Regulatory Activities v16.1. Details of AE data collection are given in eMethods. Other safety variables included changes in hematology and clinical chemistry parameters, change in 12‐lead electrocardiograms (ECGs), changes in vital sign measurements (i.e., blood pressure and pulse rate), physical and neurological examination findings, and changes in body weight, height, and calculated body mass index.

### Statistical analyses

Safety and efficacy variables were assessed for the SS and full analysis set (FAS), respectively, which each included all randomized patients who took ≥1 dose of trial medication. Analyses of all primary and secondary efficacy variables, except for seizure‐free status, included patients with ≥48 h of interpretable recordings during both the EOB and EOM video‐EEGs. For 50% responder analyses, patients who discontinued before the first 48 h of the EOM video‐EEG for reasons related to lack of efficacy were considered nonresponders; all other patients who discontinued early were classified as missing for this analysis. Analyses of seizure‐free status were performed for patients with ≥48 h of interpretable video‐EEG recording during the EOM video‐EEG.

Statistical tests of efficacy variables are presented as 2‐sided *p*‐values. Statistical comparison was performed at the 5% level of significance. Since each region had a single primary efficacy variable, adjustment for multiplicity was not needed. The ADF of electrographic focal seizures was calculated as (number of focal seizures as recorded on the video‐EEG divided by the number of interpretable hours recorded) multiplied by 24. For the US primary efficacy variable, an analysis of covariance was performed on log‐transformed ADF at EOM with terms for treatment, age category (4 age stratification categories, pooled as appropriate), and center (appropriately pooled), and log‐transformed baseline seizure ADF as a covariate. The proportion of responders was analyzed using logistic regression with terms for treatment, age category (4 age stratification categories, pooled as appropriate), and center (appropriately pooled). Sensitivity analyses of the region‐specific primary efficacy variables were conducted for the per protocol set (PPS; all patients in the FAS who did not have important protocol deviations related to efficacy), the FAS—source data verified (all patients in the FAS who had both their EOB and EOM video‐EEG electronic case report form pages source data verified using on‐site monitoring processes), and patients in the FAS who had ≥24 h of interpretable recordings (rather than ≥48 h) during both the EOB and EOM video‐EEGs. Analysis of secondary efficacy variables relied on summary statistics only.

Assuming an effect size of 0.402, power of 80%, and a 2‐sided test at the 5% level of significance, a sample of 99 patients in each treatment arm was needed. Assuming responder rates of 20% and 40% for the PBO and LCM groups, respectively, a 2‐sided continuity corrected chi‐square test at a significance level of 5% provided approximately 83% power with 99 patients in each treatment arm. To account for an anticipated difference in interpretation of the EOB video‐EEG of 5% as well as the potential patient dropout rate of approximately 14%, 122 patients per treatment arm were planned for enrollment. A blinded sample size re‐estimation was performed when 50% of patients had been randomized, completed the trial, and had data available for analysis, to check the validity of the above assumptions using interim data from the trial (see eMethods for details). This sample size re‐estimation was conducted after the requirement for the central reader was removed. Considering the low dropout rate and the low rate of difference in the interpretation of EOB video‐EEGs, the sample size re‐estimation concluded that the trial could proceed with enrollment of the originally planned sample size of 122 patients per treatment arm.

The trial protocol and statistical analysis plan are available in the trial's record on ClinicalTrials.gov (identifier: NCT02477839).

## Results

### Patient disposition, baseline demographics, and epilepsy characteristics

**Figure 2:**
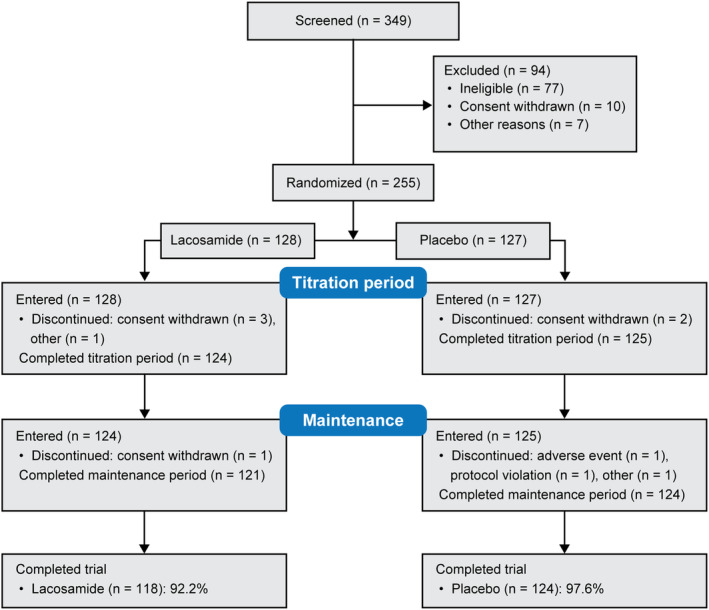
Patient disposition.

Overall, 349 patients were screened and 255 were randomized (LCM: 128; PBO: 127) (Fig. 2). All randomized patients took ≥1 dose of trial medication and were included in the SS and FAS. Most patients completed the trial (LCM: 118 [92.2%]; PBO: 124 [97.6%]). The most common reason for early discontinuation was withdrawal of consent.

Baseline demographics, epilepsy characteristics, and use of ASMs were similar in both treatment groups (Tables 1 and 2). Patients on LCM had a mean age of 2.1 years (PBO: 2.2 years), with a median epilepsy duration of 1.0 year (PBO: 1.3 years). One‐third of patients in each group had 1–3 previous ASMs (ASMs taken and stopped >14 days before visit 1 and not taken during the trial). On the day of first trial dose, 29.7% of patients on LCM and 28.3% on PBO were taking 1 ASM, 42.2% and 43.3% were taking 2 ASMs, and 26.6% and 26.8% were taking 3 ASMs. Except for 2 patients with missing data, all patients in both treatment groups used concomitant ASMs, the most common of which (≥20% in either treatment group) were valproate, levetiracetam, topiramate, and carbamazepine. Most patients had previous and ongoing medical conditions, most commonly (≥10% in either treatment group) quadriparesis, cerebral palsy, mental retardation, microcephaly, and psychomotor retardation. Over half of the patients in each treatment group received concomitant non‐ASM medications.

**Table 1:** Baseline demographics and epilepsy characteristics (safety set).

	Placebo (*n* = 127)	Lacosamide (*n* = 128)
Patient demographics		
Age, mean (SD), years	2.2 (1.1)	2.1 (1.1)
≥1 to <6 months, *n* (%)	8 (6.3)	8 (6.3)
≥6 months to <1 year, *n* (%)	13 (10.2)	18 (14.1)
≥1 to <2 years, *n* (%)	38 (29.9)	36 (28.1)
≥2 to <4 years, *n* (%)	68 (53.5)	66 (51.6)
Male, *n* (%)	75 (59.1)	71 (55.5)
Weight, mean (SD), kg	11.7 (3.4)	11.3 (3.8)
Body mass index, mean (SD), kg/m^2^	15.6 (2.1)	15.4 (2.3)
Epilepsy history		
Epilepsy duration, median (range), years	1.3 (0.1–3.7)	1.0 (0.1–3.9)
Age at diagnosis, median (range), years	0.5 (0.0–3.3)	0.4 (0.0–3.5)
Classification of seizures experienced at any point before trial entry^a^ ^,^ ^b^, *n* (%)		
Any partial‐onset seizures (focal)	127 (100)	128 (100)
Simple partial (focal aware)	47 (37.0)	56 (43.8)
Complex partial (focal impaired awareness)	94 (74.0)	82 (64.1)
Partial evolving to secondarily generalized (focal to bilateral tonic–clonic)	64 (50.4)	63 (49.2)
Any generalized seizures	9 (7.1)	11 (8.6)
Absence	0	1 (0.8)
Atypical absence	1 (0.8)	2 (1.6)
Myoclonic	3 (2.4)	0
Clonic	2 (1.6)	3 (2.3)
Tonic	4 (3.1)	8 (6.3)
Tonic–clonic	3 (2.4)	5 (3.9)
Atonic	0	2 (1.6)
Unclassified epileptic seizures	6 (4.7)	8 (6.3)
Previous and ongoing medical conditions		
Any previous and ongoing medical conditions, *n* (%)	100 (78.7)	112 (87.5)
Previous and ongoing medical conditions^c^ in ≥7% of overall population, *n* (%)		
Quadriparesis	22 (17.3)	10 (7.8)
Cerebral palsy	19 (15.0)	13 (10.2)
Mental retardation	14 (11.0)	14 (10.9)
Microcephaly	13 (10.2)	20 (15.6)
Developmental delay	9 (7.1)	12 (9.4)
Psychomotor retardation	6 (4.7)	16 (12.5)
Any concomitant non‐ASM medications^d^, *n* (%)	70 (55.1)	68 (53.1)

^a^ Seizure types are listed per the trial protocol (International League Against Epilepsy [ILAE] 1981 [Epilepsia 1981;22:489–501]) with the ILAE 2017 classification (Fisher et al. Epilepsia 2017;58:522–530) provided in parentheses.

^b^ Multiple seizure types could be reported.

^c^ Medical Dictionary for Regulatory Activities v16.1 Preferred Term.

^d^ Medications taken concomitantly for ≥1 day in common with trial medication.

**Table 2:** Antiseizure medications (safety set).

	Placebo (*n* = 127)	Lacosamide (*n* = 128)
Number of previous ASMs^a^, *n* (%)		
0	74 (58.3)	81 (63.3)
1–3	45 (35.4)	44 (34.4)
4–6	8 (6.3)	3 (2.3)
≥7	0	0
Number of ASMs taken on the day of first trial dose, *n* (%)		
1	36 (28.3)	38 (29.7)
2	55 (43.3)	54 (42.2)
3	34 (26.8)	34 (26.6)
≥4	1 (0.8)	1 (0.8)
Missing	1 (0.8)	1 (0.8)
Concomitant ASMs^b^		
Any concomitant ASMs, *n* (%)	126 (99.2)	127 (99.2)
Concomitant ASMs taken by ≥10% of overall population, *n* (%)		
Valproate^c^	71 (55.9)	57 (44.5)
Levetiracetam	54 (42.5)	60 (46.9)
Topiramate	31 (24.4)	24 (18.8)
Clobazam	15 (11.8)	12 (9.4)
Carbamazepine	13 (10.2)	28 (21.9)
Oxcarbazepine	13 (10.2)	15 (11.7)

ASM, antiseizure medication.

^a^ ASMs taken and stopped >14 days before visit 1 (i.e., before entry into the baseline period), and not taken during the trial.

^b^ ASMs taken concomitantly for ≥1 day in common with trial medication.

^c^ Category includes valproic acid, valproate semisodium, valproate sodium, ergenyl chrono, and valpromide.

The median duration of exposure to trial medication during the treatment period was similar in the LCM (27.0 [range 3–44] days) and PBO groups (27.0 [6–32] days) (SS). The mean of the median total daily dose was 9.1 mg/kg/day and 9.2 mg/kg/day for the LCM and PBO groups, respectively.

### Efficacy

**Figure 3:**
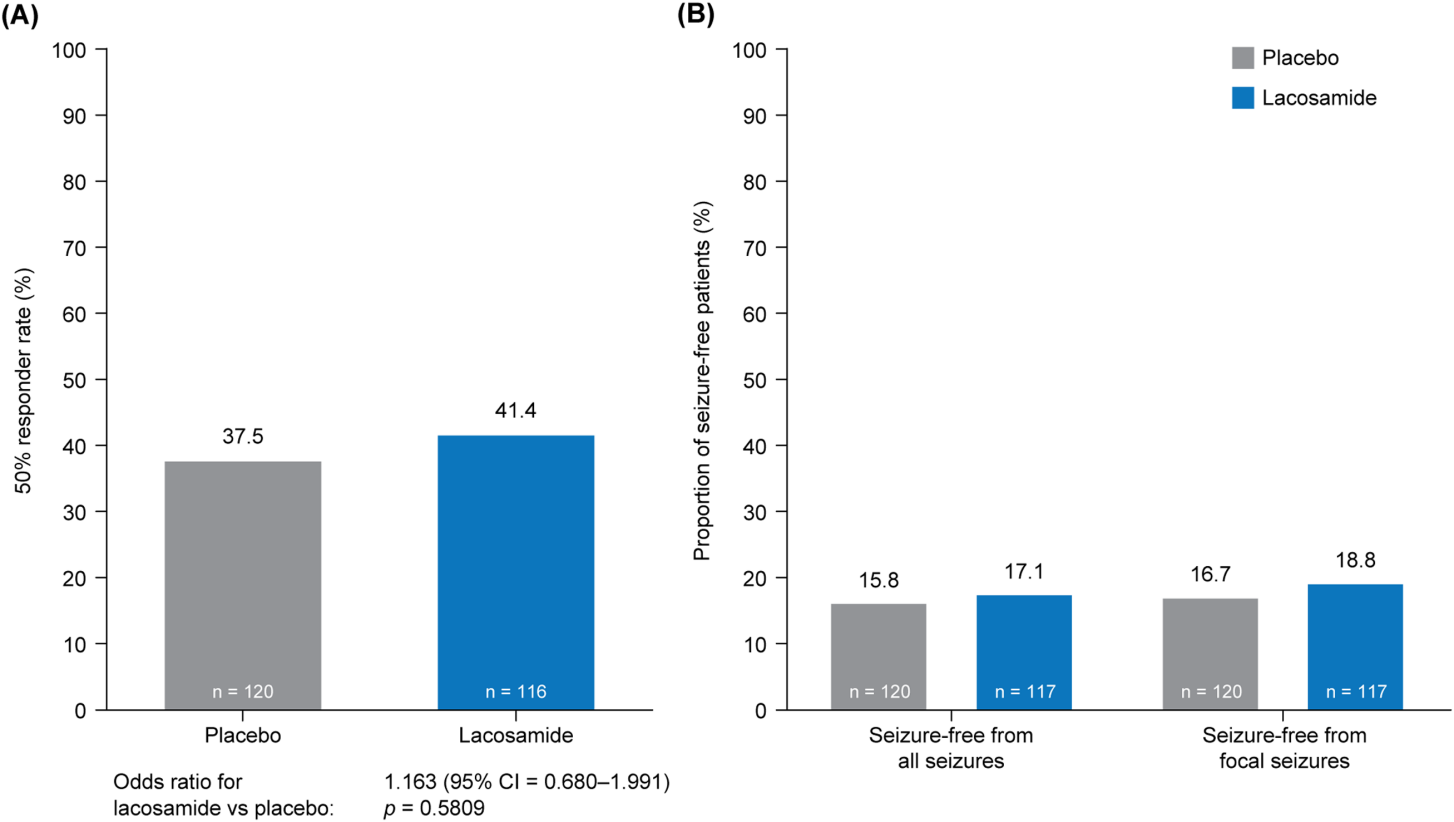
Analyses of (A) change in 50% responder rate^a^ from the EOB to the EOM and (B) seizure‐free status^b^ (FAS). ADF, average daily frequency; CI, confidence interval; EOB, end‐of‐baseline period; EEG, electroencephalogram; EOM, end‐of‐maintenance period; FAS, full analysis set. ^a^The 50% responder rate was the proportion of patients who experienced a ≥50% reduction in ADF of electrographic focal seizures recorded on the EOM video‐EEG compared with the EOB video‐EEG. Patients with 0 seizures on the EOB video‐EEG were considered nonresponders. The analysis consists of all patients in the FAS who had ≥48 h of interpretable recording during both the EOB and the EOM video‐EEGs. Patients who had ≥48 h of interpretable recording during the EOB video‐EEG, but who discontinued the trial before the first 48 h of the EOM video‐EEG for reasons related to lack of efficacy, were considered nonresponders and were included in the analysis, whereas all other patients were excluded from the analysis. Note that no patients in the trial discontinued due to lack of efficacy. Odds ratio, 95% CI, and *p*‐value were from a logistic regression model with factors for treatment, pooled randomized age stratum, and pooled center. ^b^Percentages were based on the number of patients in the FAS who completed ≥48 h of interpretable recording during the EOM video‐EEG. A patient was considered seizure‐free from all seizures if the EOM video‐EEG had 0 seizures reported. A patient was considered seizure‐free from focal seizures if the EOM video‐EEG had 0 focal seizures reported.

The median ADF of focal seizures from the EOB video‐EEG was 3.6 for the LCM group and 4.0 for the PBO group (Table 3). For the US primary efficacy variable, the percentage reduction in ADF of focal seizures for LCM versus PBO was 3.2% (95% confidence interval = −13.6 to 17.5), which was not statistically significant (*p* = 0.69). The median percentage change in ADF of focal seizures from EOB to EOM (secondary efficacy variable) was −40.2% for LCM versus −32.0% for PBO (median absolute change: −1.4 vs −1.0, respectively). The EU primary efficacy variable, the 50% responder rate during the maintenance period, was numerically higher with LCM than PBO (41.4% vs 37.5%), but the difference was not statistically significant (*p* = 0.58) (Fig. 3A). For both region‐specific primary efficacy variables, the results of sensitivity analyses conducted on the PPS, FAS—source data verified, or patients in the FAS who had ≥24 h of interpretable recordings (rather than ≥48 h) during both the EOB and EOM video‐EEGs were consistent with those for the overall analyses.

**Table 3:** Change in ADF of electrographic focal seizures, in patients with ≥48 h of interpretable video‐EEG recording (full analysis set).

	Placebo (*n* = 120)	Lacosamide (*n* = 116)
ADF of electrographic focal seizures		
EOB, median (range)	4.0 (0–246.9)	3.6 (0.7–153.8)
EOM, median (range)	2.8 (0–97.6)	2.0 (0–98.8)
Change from EOB to EOM in ADF, median (range)		
Absolute change	−1.0 (−173.8 to 14.3)	−1.4 (−55.0 to 13.0)
Percentage change	−32.0 (−100.0 to 200.8)	−40.2 (−100.0 to 347.6)
Analysis of change in ADF of electrographic focal seizures as measured on the EOM video‐EEG compared with the EOB video‐EEG		
LS mean (SE)	1.4 (0.1)	1.4 (0.1)
Difference vs placebo (95% CI)	–	1.0 (0.8–1.1)
Percentage reduction vs placebo (95% CI)	–	3.2 (−13.6 to 17.5)
*p*‐value	–	0.69

ADF, average daily frequency; ANCOVA, analysis of covariance; CI, confidence interval; EEG, electroencephalogram; EOB, end‐of‐baseline period; EOM, end‐of‐maintenance period; LS, least‐squares; SE, standard error. The analysis consisted of all patients in the full analysis set who had ≥48 h of interpretable recording during both the EOB and the EOM video‐EEGs. Focal seizure frequency was based on electrographic seizures for infants aged ≥1 to ≤6 months, and on electrographic seizures with an accompanying clinical correlate for children aged >6 months to <4 years. Seizure frequency was analyzed using an ANCOVA with terms for treatment, pooled randomized age stratum, pooled center, and baseline seizure ADF. Seizure ADF was log‐transformed using the transformation of ln(X + 1), where X was the seizure ADF. Baseline seizure ADF was log‐transformed. LS means were based on log‐transformed data of the full ANCOVA model. Difference ratio in LS mean was calculated as the exp[LS mean lacosamide − LS mean placebo]. Percentage reduction over placebo was estimated as 100 × (1 − exp[LS mean lacosamide − LS mean placebo]).

**Figure 4:**
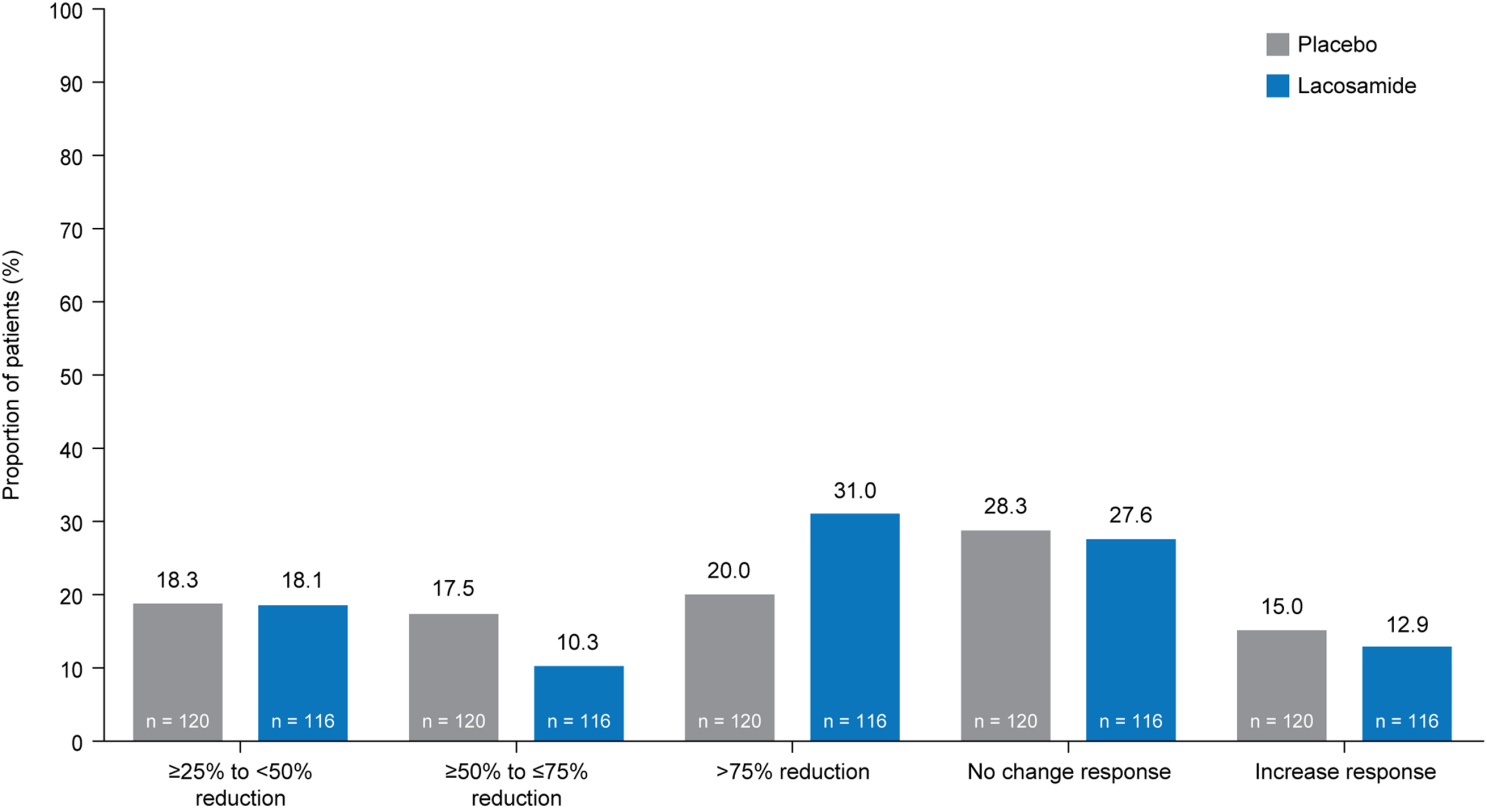
Proportion of patients who experienced a reduction, no change response, or increase response in ADF of electrographic focal seizures from the EOB to the EOM^a^ (full analysis set). ADF, average daily frequency; EEG, electroencephalogram; EOB, end‐of‐baseline period; EOM, end‐of‐maintenance period. ^a^Percentages were based on the number of patients in the full analysis set who had ≥48 h of interpretable recording during both the EOB and the EOM video‐EEGs. A ≥25% to <50% response was defined as a ≥25% to <50% reduction in ADF of electrographic focal seizures from the EOB video‐EEG to the EOM video‐EEG. A ≥50% to ≤75% response was defined as a ≥50% to ≤75% reduction in ADF of electrographic focal seizures from the EOB video‐EEG to the EOM video‐EEG. A >75% response was defined as a >75% reduction in ADF of electrographic focal seizures from the EOB video‐EEG to the EOM video‐EEG. No change was defined as between a <25% reduction and <25% increase in ADF of electrographic focal seizures from the EOB video‐EEG to the EOM video‐EEG. An increase was defined as a ≥25% increase in ADF of electrographic focal seizures from the EOB video‐EEG to the EOM video‐EEG.

Few patients achieved seizure‐free status either for all seizure types or focal seizures (Fig. 3B). The proportion of patients with a ≥25% to <50% reduction in ADF of electrographic focal seizures was similar in both treatment groups (LCM: 18.1%; PBO: 18.3%), the proportion of patients with a ≥50% to ≤75% reduction was numerically lower with LCM (10.3%; 17.5%), and the proportion of patients with a >75% reduction was numerically higher with LCM (31.0%; 20.0%) (Fig. 4). The proportions of patients who experienced “no change” (LCM: 27.6%; PBO: 28.3%) or an increase in ADF of electrographic focal seizures (12.9%; 15.0%) were similar in both treatment groups.

Assessments of Clinical GIC at EOM favored LCM, with 68.0% of patients experiencing an improvement (i.e., very much, much, or minimally improved) versus 59.8% on PBO (Fig. S1A). The largest difference was seen in the “much improved” category (LCM: 33.6%; PBO: 21.3%). Caregiver's GIC also favored patients on LCM, as 76.6% versus 61.4% on PBO experienced an improvement (Fig. S1B). The largest difference was again seen in the “much improved” category (LCM: 32.8%; PBO: 19.7%).

A favorable trend of larger mean changes from baseline to EOM in PedsQL total and subscale scores was observed in patients aged 2–4 years on LCM versus PBO, but these changes were small and associated with large standard deviations (Table S1). The associated median changes for LCM were 0 for all but the total and psychosocial health summary scores (1.9 and 2.5, respectively), and did not show clear differentiation over PBO.

### Safety

During the treatment period, treatment‐emergent AEs (TEAEs) were reported by 44.5% of patients on LCM and 51.2% on PBO (Table 4). The incidence of TEAEs considered drug‐related by the investigator was 22.7% with LCM and 11.8% with PBO. In the LCM group, the most common TEAEs (≥5% of patients) were somnolence (11.7%), irritability (5.5%), and vomiting (5.5%); and the most common drug‐related TEAE (≥5% of patients) was somnolence (10.9%). In both groups, TEAEs and drug‐related TEAEs were more common during titration than maintenance. Four patients reported serious TEAEs in each treatment group. Serious TEAEs with LCM were vomiting (2 [1.6%]) and convulsion (2 [1.6%]); serious TEAEs with PBO were pyrexia, respiratory failure, thermal burn, upper respiratory tract infection, and urinary tract infection (1 [0.8%] patient each). The 2 events of vomiting with LCM were considered drug‐related and occurred during the titration period in a 2‐year‐old female and a 2‐year‐old male.

**Table 4:** TEAEs during the titration, maintenance, and treatment periods (safety set).

Patients, *n* (%)	Titration period		Maintenance period		Treatment period (titration + maintenance)	
	Placebo (*n* = 127)	Lacosamide (*n* = 128)	Placebo (*n* = 125)	Lacosamide (*n* = 124)	Placebo (*n* = 127)	Lacosamide (*n* = 128)
Any TEAEs	45 (35.4)	45 (35.2)	36 (28.8)	28 (22.6)	65 (51.2)	57 (44.5)
Serious TEAEs	1 (0.8)	4 (3.1)	3 (2.4)	0	4 (3.1)	4 (3.1)
Discontinuations due to TEAEs	0	0	0	2 (1.6)	0	2 (1.6)
Drug‐related TEAEs^a^	13 (10.2)	26 (20.3)	4 (3.2)	5 (4.0)	15 (11.8)	29 (22.7)
Drug‐related serious TEAEs^a^	0	2 (1.6)	0	0	0	2 (1.6)
Severe TEAEs	0	1 (0.8)	2 (1.6)	2 (1.6)	2 (1.6)	3 (2.3)
All deaths (AEs leading to death)	0	0	0	0	0	0
Deaths (TEAEs leading to death)	0	0	0	0	0	0
TEAEs^b^ reported by ≥5% of patients in either treatment group						
Somnolence	3 (2.4)	13 (10.2)	1 (0.8)	2 (1.6)	4 (3.1)	15 (11.7)
Irritability	2 (1.6)	6 (4.7)	3 (2.4)	1 (0.8)	5 (3.9)	7 (5.5)
Vomiting	4 (3.1)	7 (5.5)	1 (0.8)	1 (0.8)	4 (3.1)	7 (5.5)
Pyrexia	9 (7.1)	4 (3.1)	5 (4.0)	3 (2.4)	14 (11.0)	6 (4.7)
Upper respiratory tract infection	10 (7.9)	2 (1.6)	2 (1.6)	1 (0.8)	11 (8.7)	3 (2.3)

AE, adverse event; TEAE, treatment‐emergent adverse event.

^a^ Related TEAEs were determined as per the investigator.

^b^ Medical Dictionary for Regulatory Activities v16.1 Preferred Term.

Two patients on LCM discontinued because of TEAEs. A 1‐year‐old male discontinued because of idiopathic generalized epilepsy that occurred during the maintenance period, with a dose at onset of 10 mg/kg/day; the event was considered mild in intensity. A 2‐year‐old female discontinued because of sinus bradycardia, which occurred during the maintenance period at a dose at onset of 6 mg/kg/day; the event was considered mild in intensity and was ongoing at the time of discontinuation.

At baseline, over 80% of patients in each treatment arm had a normal ECG interpretation, whereas post baseline, 39.8% of patients on LCM and 33.0% on PBO had ≥1 abnormal, nonclinically significant ECG result. One patient, in the PBO group, had a post‐baseline abnormal and clinically significant ECG result. No consistent or clinically relevant changes from baseline in vital sign parameters were observed, and no consistent or clinically relevant changes from baseline after treatment onset were observed in mean hematology or clinical chemistry values that were considered related to LCM. The incidence of shifts in neurologic examination findings from normal at baseline to abnormal and clinically significant at post‐baseline visits was low overall and similar for both treatment groups.

## Discussion

LCM was recently approved for the treatment of focal seizures in children aged ≥1 month in the US and ≥2 years in the EU, based on the extrapolation of data from adults.^4^, ^5^, ^7^ However, at the time SP0967 was initiated, it was still necessary to establish the efficacy and safety of LCM in patients younger than 4 years in a randomized controlled trial. This double‐blind, PBO‐controlled trial in patients aged ≥1 month to <4 years with uncontrolled focal seizures did not show superior efficacy of LCM versus PBO administered concomitantly with 1–3 ASMs, as assessed by the change in ADF of focal seizures and the 50% responder rate based on video‐EEG. The trial faced multiple challenges, and methodological issues may have influenced the results.^6^

Recruitment to PBO‐controlled trials using video‐EEG is difficult in very young children for various reasons, such as the risk of being randomized to PBO when the trial drug is usually available for off‐label prescription, and the requirement to undergo 2 inpatient video‐EEGs, each lasting ≥48 h.^8^ As LCM was commercially available from the beginning of SP0967 (indicated for use in patients aged ≥17 years in the US and ≥16 years in the EU), it was difficult to find eligible children who were not already receiving LCM off‐label in many countries. Recruitment to the youngest age group (≥1 to <6 months) was very difficult, whereas the other age groups reached the targets more quickly. It was decided to stop the trial when the planned overall number of patients was reached. Therefore, the numbers of patients in different age groups and randomization age strata differed slightly from the protocol‐defined targets. The ≥1 to <6 months age group included fewer patients than planned (16 rather than ≥25), whereas the remaining 3 age groups met the protocol‐defined targets (≥25 patients in the ≥6 months to <1 year and ≥1 to <2 years groups; ≥20 patients in the ≥2 to <4 years group). Despite this, SP0967 recruited a higher number of patients aged <1 year (47 patients) compared with similar trials in levetiracetam (23 patients)^9^ and pregabalin (18 patients).^10^

A further challenge was the requirement for children to undergo ≥48 and ≤72 h of video‐EEG twice during the trial. Young children have a low tolerability for video‐EEGs lasting extended periods, and the observation period was far longer than in routine practice, as 24 h (48 h in exceptional circumstances) is generally sufficient to define the type of frequent seizures in a hospital setting. Counting of seizures during video‐EEG is also unusual in routine practice. These requirements challenged both families and site personnel, bound their resources significantly, and led to many refusals of participation.

Issues with using video‐EEG as a measure of efficacy may also have been a factor in the negative results of SP0967, as high variability and low reliability was found between readers in the interpretation of video‐EEGs, as reported in the companion paper.^6^ The planned sample size accounted for an anticipated difference in interpretation of the EOB video‐EEG of 5% between the central reader and local investigators. However, a review of the first group of 7 EOB video‐EEGs by the central reader showed that for 71% of the patients that local investigators randomized after considering the EOB video‐EEG to show ≥2 focal seizures, the central reader did not count ≥2 focal seizures (the count was typically 0), that is, the rate of eligible patients for efficacy analyses was around 29%, rather than the 95% assumed by the protocol.^6^ This led to the decision to remove the central reader and allocate the responsibility for assessing seizure counts for the efficacy analyses to the investigators partway through the trial, after discussion with the regulatory agencies. After this change to the protocol, the difference in interpretation rate became <5%. Problems with using video‐EEG to measure efficacy in trials involving infants and young children are known, and there is a proposal for an alternative design to the traditional PBO‐controlled trials for studying new ASMs to treat focal seizures in children aged 1 month to 4 years.^8^ The new design employs seizure counting by caregivers based on previous video‐EEG/video validation of specific seizure semiologies, with “time to Nth seizure” as the primary outcome, and incorporates variable baseline duration.

In the current trial, there was generally little difference between LCM and PBO groups in the primary and secondary efficacy variables. Both groups showed substantial reductions in ADF of focal seizures. Caregiver's GIC, a variable not based on video‐EEG, showed a general trend toward a larger improvement for LCM versus PBO. The discrepancy between the results for the primary and secondary efficacy variables and Caregiver's GIC may be explained by the fact that GIC is a subjective scale. Also, video‐EEG measures only one aspect of epilepsy, namely seizures; improvement in other comorbid symptoms observed in children with epilepsy (e.g., behavior) may explain this discordance. However, this is speculative as we only have global impression and no detailed data.

LCM was generally well tolerated and demonstrated an acceptable safety profile, with similar incidences of TEAEs, serious TEAEs, and discontinuations because of TEAEs compared with PBO. The safety findings were consistent with the known safety profile of LCM in adults^11^, ^12^, ^13^ and children aged ≥4 years,^14^ and were as expected for this pediatric population. The most common TEAEs with LCM were in line with the US Prescribing Information and EU Summary of Product Characteristics.^4^, ^5^

Previous studies have demonstrated the efficacy of adjunctive LCM in older children^14^ and adults with focal seizures.^11^, ^13^, ^15^ The efficacy of LCM in children and adolescents (aged ≥4 to <17 years) with uncontrolled focal seizures was established in a double‐blind, PBO‐controlled trial (SP0969: NCT01921205).^14^ Efficacy variables were based on caregiver‐completed seizure diaries rather than video‐EEGs. The primary efficacy variable of change in focal seizure frequency per 28 days from baseline to maintenance showed a percentage reduction of 31.7% for LCM versus PBO (*p* = 0.0003). The median percentage reduction in focal seizure frequency per 28 days during maintenance was 51.7% for LCM compared with 21.7% for PBO. The 50% responder rate was also higher for LCM versus PBO (52.9% vs 33.3%, odds ratio 2.17, *p* = 0.0006).

Given the results of SP0969, there was no reason to suspect that LCM would not also be effective in younger children with focal seizures. While this trial did not demonstrate the efficacy of LCM versus PBO, differences in the patient population, design (including endpoint utilized), and variables of the current trial and SP0969 may have led to their different outcomes. Both trials enrolled patients with focal seizures, but the current trial included a higher proportion of patients who also had generalized (7.8% vs 1.5% in SP0969^14^) or unclassified epileptic seizures (5.5% vs 0.6% in SP0969^14^). Three (2.4%) patients in this trial had myoclonic seizures. The notion that the enrolled patients were not a pure population with only focal seizures is consistent with quadriparesis, cerebral palsy, mental retardation, microcephaly, developmental delay, and psychomotor retardation being the most prevalent comorbid medical conditions, as these are common comorbid conditions in epilepsy syndromes such as Lennox–Gastaut syndrome. Although both studies were randomized, double‐blind, PBO‐controlled trials with body weight‐adjusted dosing, the current trial had a much shorter treatment period than SP0969 (20‐day titration and 7‐day maintenance vs 6‐week titration and 10‐week maintenance^14^). Additionally, baseline seizure frequency was assessed during ≤72 h of video‐EEG in the current trial versus 8‐week prospective baseline in SP0969.^14^ Given the short trial duration and maintenance period, day‐to‐day variability in seizure counts may have partly contributed to the different outcomes versus SP0969. Finally, the different methodologies used to assess efficacy (video‐EEG vs caregiver‐completed seizure diaries) may have contributed to the differing outcomes of the 2 trials.

## Conclusions

In this double‐blind, PBO‐controlled trial, adjunctive LCM did not show superior efficacy compared with PBO in patients aged ≥1 month to <4 years with uncontrolled focal seizures. However, primary and secondary efficacy variables were potentially affected by high variability and low reliability between readers in video‐EEG interpretation. LCM was generally well tolerated, with an acceptable safety profile consistent with the known safety profile of LCM in adults and children aged ≥4 years.

## Funding Information

This trial was supported by UCB Pharma (Brussels, Belgium), which was involved in the design of the trial; the collection, analysis, and interpretation of data; and in the decision to publish the manuscript. Several authors (C. Beller, J. Csikós, C. McClung, and H. Moeltgen) are employees of UCB Pharma. A. Bozorg was employed by UCB Pharma at the time of the trial.

## Author Contributions

I.M., Y.‐T.N., and M.K.F. contributed to acquisition of data, analysis or interpretation of data, and drafting/revising the manuscript for content. C.B. contributed to trial concept or design, analysis or interpretation of data, statistical analyses, and drafting/revising the manuscript for content. A.B., J.C., C.M., and H.M. contributed to trial concept or design, analysis or interpretation of data, and drafting/revising the manuscript for content.

## Conflict of Interest

I.M. and Y.‐T.N. have nothing to report. C.B., J.C., C.M., and H.M. are employees of UCB Pharma. A.B. was employed by UCB Pharma at the time of the trial, and is currently employed by Otsuka Pharmaceutical Development & Commercialization, Inc. C.B. and J.C. have received UCB Pharma stocks from their employment. A.B. owned UCB Pharma stocks during the conduct of the trial. C.M. and H.M. have received UCB Pharma stocks/stock options from their employment. M.K.F. has received payment or honoraria for lectures, presentations, speakers bureaus, manuscript writing, or educational events from BioMarin, Nutricia Hungary, and PTC Therapeutics, and support for attending meetings and/or travel from BioMarin and PTC Therapeutics.

## Supporting information


Appendix S1.

## Data Availability

Underlying data from this manuscript may be requested by qualified researchers 6 months after product or indication approval in the US and/or Europe, or global development is discontinued, and 18 months after trial completion. Investigators may request access to anonymized individual patient‐level data and redacted trial documents, which may include: analysis‐ready datasets, study protocol, annotated case report form, statistical analysis plan, dataset specifications, and clinical study report. Before use of the data, proposals need to be approved by an independent review panel at www.Vivli.org and a signed data sharing agreement will need to be executed. All documents are available in English only, for a prespecified time, typically 12 months, on a password protected portal.
