# Supplementary material for: Efficacy and tolerability of adjunctive lacosamide in patients aged <4 years with focal seizures
Source: Ann Clin Transl Neurol. 2024 Feb 20;11(3):768–79. doi: 10.1002/acn3.52004 (PMC10963285; doi:10.1002/acn3.52004)
Supplement: Supplementary file 1 — Appendix S1. [file ACN3-11-768-s001.pdf]

Supplementary Materials

eMethods

Additional Exclusion Criteria

Patients were excluded if they were on a ketogenic diet that had either changed within the previous 4 weeks or was expected to change during the trial; creatinine clearance of <30 mL/minute; clinically relevant electrocardiogram abnormality; hemodynamically significant congenital heart disease; arrhythmic heart condition requiring medical therapy; history of severe anaphylactic reaction secondary to medication intake or serious blood dyscrasias; nonepileptic events that could be confused with seizures; serious toxicity issues while on felbamate treatment or felbamate treatment for <12 months; acute or subacutely progressive central nervous system disease, or epilepsy secondary to a progressive cerebral or neurodegenerative disease; or cardiac sodium channelopathy.

Protocol Amendments

The inclusion and exclusion criteria described in this manuscript reflect those used in the last version of the trial protocol. During the conduct of the trial, certain criteria were removed or adjusted in protocol amendments to provide additional clarity, make the protocol more patient-friendly, and enhance enrollment. All protocol amendments were implemented before unblinding. Important amendments included the following:

- Removal of the inclusion criterion requiring patients to have uncontrolled focal seizures after an adequate course of treatment with ≥2 antiseizure medications (ASMs). The criterion was removed because there was another inclusion criterion that targeted the population of interest with regard to concomitant ASMs, and because requiring patients to fail ≥2 ASMs was an impediment to recruiting younger patients.
- Change of the duration of video-electroencephalograms (video-EEGs) from 72 hours of continuous recording to ≤72 hours of continuous recording, with every attempt to obtain ≥48 hours of interpretable recording. The rationale was that completion of a full 72-hour EEG was an impediment to patient enrollment.

- Removal of the central video-EEG reader, which was applied to address the high variability in video-EEG seizure counts between the central reader and local investigators (Bozorg A et al, 2024).

Randomization of Patients

An interactive voice/web response system (IXRS) was used to assign a treatment to eligible patients, based on a predetermined randomization schedule provided by UCB Pharma or its designee. Patients, investigators, and all site personnel were blinded to trial medication. To enroll a patient (visit 1), the investigator contacted the IXRS. Each patient was assigned a number at screening that served as the patient identifier throughout the trial. To randomize a patient (visit 3), the investigator (or designee) contacted the IXRS. The IXRS allocated kit numbers to the patient (based on the patient number) during the course of the trial. The blind was maintained, as all patient treatment details were allocated and maintained by the IXRS and the accompanying packaging for lacosamide and placebo was identical in appearance.

Sample Size Re-estimation

The sample size re-estimation with equal allocation in each arm was performed using the related residual variance combined with Guenther adjustment (Friede and Kieser, 2011). An assessment of the observed dropout rate was also made as part of this analysis, and the potential dropout rate of 14% used for calculation of the initial sample size was modified based on the observed rate. Additionally, an assessment of the observed difference of end-of-baseline (EOB) video-EEG interpretation rate was made; the anticipated rate of 5% was modified based on the observed rate.

The final sample size, the anticipated dropout rate, and the anticipated rate of difference of interpretation of the EOB video-EEG was to be modified according to the sample size re-estimate; however, an upper bound was applied to reach a maximum sample size based on practical and logistical considerations. The initial sample size re-estimate using Guenther

adjustment was not to be adjusted above 109 patients per treatment arm, the original estimated overall trial dropout rate was not to be adjusted above 24%, and the difference of EOB video-EEG interpretation rate was not to be adjusted above 10%.

Criteria for Patient Withdrawal From the Trial

Patients must have been withdrawn from the trial if any of the following events occurred:

- The patient experienced intolerable adverse events that, in the investigator's opinion, precluded further participation in the trial. Patients who were unable to tolerate the trial medication (unable to achieve or maintain the target dose for the final 3 days of the titration period) also had to be withdrawn from the trial.
- The sponsor or a regulatory agency requested withdrawal of the patient.
- The patient had QTc interval ≥500 ms, confirmed by a cardiologist over-read on any electrocardiogram.
- The patient developed a second- or third-degree atrioventricular block.
- The patient was unwilling or unable to continue, or the parent(s)/legal representative(s)/caregiver(s) was/were unwilling or unable to allow the patient to continue in the trial.
- Investigator's decision that withdrawal from further participation would be in the patient's best interest.
- The patient experienced generalized convulsive status epilepticus.

Patients may have been withdrawn from the trial if any of the following events occurred:

- The patient had any clinically relevant change in medical or psychiatric condition and, in the investigator's opinion, the condition warranted discontinuation from the trial.
- The patient required a medication not permitted by the protocol.
- The patient's legal representative(s)/caregiver(s) (in accordance with local regulation) was/were noncompliant with trial procedures or medication, in the investigator's opinion.

- The patient used benzodiazepine within 24 hours before or during video-EEG for any reason other than a stable daily dosage regimen as a concomitant ASM.

Additional discontinuation criteria for potential drug-induced liver injury (PDILI):

- Patients with PDILI were assessed to determine if the trial medication was to be discontinued.
- All concomitant medications and herbal supplements that were not medically necessary were also to be discontinued.
- The following PDILI criteria required immediate and permanent discontinuation of trial medication:
    - Patients with either of the following:
        - Alanine aminotransferase (ALT) or aspartate aminotransferase (AST) ≥5 × upper limit of normal (ULN)
        - ALT or AST ≥3 × ULN and coexisting total bilirubin ≥2 × ULN
- The following PDILI criteria required immediate discontinuation of trial medication:
    - Patients with ALT or AST ≥3 × ULN who exhibited temporally associated symptoms of hepatitis or hypersensitivity.
- The following PDILI criteria allowed patients to continue on trial medication, at the investigator's discretion:
    - Patients with ALT or AST ≥3 × ULN (and ≥2 × baseline) and <5 × ULN, total bilirubin <2 × ULN, and no eosinophilia (i.e., ≤5%), with no fever, rash, or symptoms of hepatitis.

Collection of Adverse Event Data

All adverse events (AEs) that occurred during the study (i.e., after the signing of the informed consent form) were reported in the electronic case report form, even if no investigational product was taken but specific study procedures were conducted. This included all AEs not present before the initial visit and all AEs that recurred or worsened after the initial visit. Signs

or symptoms of the condition for which the investigational product was being studied were recorded as AEs only if their nature changed considerably or their frequency or intensity increased in a clinically significant manner compared with the clinical profile known to the investigator from the study participant's history or the baseline period.

An increase in the intensity of an AE led to the repetition of the AE being reported with either the outcome date of the first AE that was not related to the natural course of the disease being the same as the start date of the repeated AE, and the outcome of "worsening"; or the AE verbatim term being the same for the first and repeated AEs, so that the repeated AE could be easily identified as the worsening of the first one.

The relatedness of AEs was assessed by the investigators. The intensity of AEs was graded as mild, moderate, or severe based on the grades defined in Common Terminology Criteria for Adverse Events v5.0 (grade 1: mild; asymptomatic or mild symptoms; clinical or diagnostic observations only; or intervention not indicated; grade 2: moderate; minimal, local, or noninvasive intervention indicated; or limiting age-appropriate instrumental activities of daily living; grade 3: severe or medically significant but not immediately life-threatening; hospitalization or prolongation of hospitalization indicated; disabling; or limiting self-care activities of daily living; grade 4: life-threatening consequences or urgent intervention indicated; grade 5: death related to AE). Life-threatening and death due to AE was used additionally.

References

Bozorg A, Beller C, Jensen L, et al. Pitfalls of using video-EEG for a trial endpoint in children aged <4 years with focal seizures. ACN3. 2024;11:780-790.

Friede T, Kieser M. Blinded sample size recalculation for clinical trials with normal data and baseline adjusted analysis. Pharm Stat. 2011;10:8-13.

eFIGURE 1: Clinical Global Impression of Change (A) and Caregiver's Global Impression of Change (B) at EOM (Full Analysis Set).

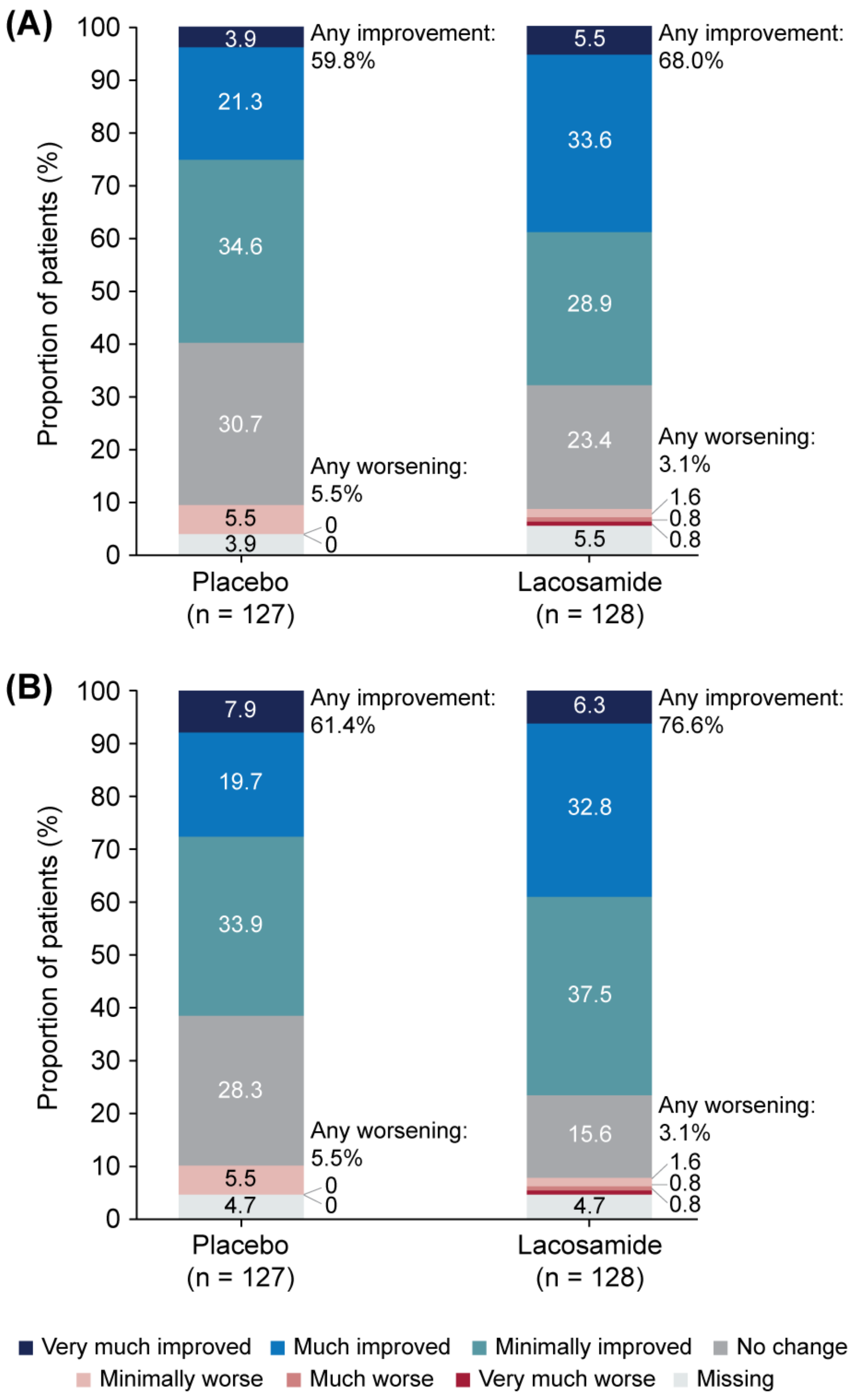

Abbreviations: EOM = end-of-maintenance period.

eTABLE 1. Change From Baseline to EOM in PedsQL Subscale Scores and Total Score for Patients Aged 2–4 Years (Full Analysis Set)

	Observed value at baseline		Change from baseline to the end of the maintenance period	
	**Placebo (n = 68)**	**Lacosamide (n = 66)**	**Placebo (n = 68)**	**Lacosamide (n = 66)**
**Physical functioning**				
n	67	65	64	63
Mean (SD)	54.9 (33.7)	52.7 (32.2)	−0.8 (18.0)	2.7 (15.8)
**Emotional functioning**				
n	67	65	64	62
Mean (SD)	68.5 (22.7)	63.4 (23.2)	1.3 (19.4)	3.4 (16.7)
**Social functioning**				
n	66	65	63	62
Mean (SD)	59.1 (31.6)	60.2 (32.0)	0.7 (23.3)	2.0 (22.2)
**School functioning**				
n	30	37	27	30
Mean (SD)	66.9 (30.3)	51.6 (33.7)	0.3 (22.9)	4.2 (27.9)
**Psychosocial health summary score**				
n	66	65	63	61
Mean (SD)	63.8 (21.9)	60.3 (23.9)	1.1 (15.3)	3.2 (15.1)
**Physical health summary score**				
n	67	65	64	63
Mean (SD)	54.9 (33.7)	52.7 (32.2)	−0.8 (18.0)	2.7 (15.8)
**Total score**				
n	67	65	64	62
Mean (SD)	59.5 (24.8)	57.0 (25.1)	0.3 (12.7)	2.7 (14.2)

Abbreviations: EOM = end-of-maintenance period; PedsQL = Pediatric Quality of Life Inventory; SD = standard deviation.

Appendix 1

Investigators

Name	Location	Role	Contribution
**Dr Conceição Campanário da Silva Pereira Almeida**	Hospital Infantil Sabará, São Paulo, Brazil	Site Investigator	Data acquisition
**Dr Anna Altmann**	Servus Salvus Egészségügyi Szolgáltató Kft, Budapest, Hungary	Site Investigator	Data acquisition
**Dr Elena Arefieva**	State Autonomous Healthcare Institution of the Kemerovo Region “Kemerovo Regional Clinical Hospital named after S.V. Belyaev”, Kemerovo, Russia	Site Investigator	Data acquisition
**Dr Susan Arnold**	Children’s Medical Center Dallas, Dallas, TX, USA	Site Investigator	Data acquisition
**Dr Mihaela Axente**	Centrul National Clinic De Recuperare Neuropsihomotorie pentru Copii - Dr N. Robanescu, Bucharest, Romania	Site Investigator	Data acquisition
**Dr Maria Giuseppina Baglietto**	IRCCS Istituto Giannina Gaslini, Unitá Operativa di Neuropsichiatria Infantile, Genova, Italy	Site Investigator	Data acquisition
**Prof. Sophio Bakhtadze**	Tbilisi State Medical University, G. Zhvania Academic Clinic of Pediatrics, Tbilisi, Georgia	Site Investigator	Data acquisition

**Dr Domenica Immacolata Battaglia**	Fondazione Policlinico Universitario Agostino Gemelli, UOC Neuropsichiatria Infantile, Rome, Italy	Site Investigator	Data acquisition
**Prof. Liudmila Belova**	State Health Institution “Central Clinical Medical Sanitary Department named after the Honored Doctor of Russia V.A. Egorova”, Ulianovsk, Russia	Site Investigator	Data acquisition
**Dr Marianne Berényi**	Szent Margit Kórház, Fejlődésneurológiai Osztály, Budapest, Hungary	Site Investigator	Data acquisition
**Dr Cornelia Calcii**	Institutia Medico-Sanitara Publica (IMSP), Institutul Mamei si Copilului, Sectia Neurologie si Epileptologie, Chisinau, Moldova	Site Investigator	Data acquisition
**Dr Dezhi Cao**	Shenzhen Children’s Hospital, Department of Pediatric Neurology, Shenzhen, China	Site Investigator	Data acquisition
**Dr Juan Fernando Capristo Gonzales**	Centenario Hospital Miguel Hidalgo, Aguascalientes, Mexico	Site Investigator	Data acquisition
**Dr Hugo Ceja Moreno**	Antiguo Hospital Civil de Guadalajara Fray Antonio Alcalde, Hospital n° 278, Tercer Piso Neuropediatría, Colonia El Retiro, Guadalajara, Mexico	Site Investigator	Data acquisition
**Assoc. Prof. Yuriy Chomolyak**	Regional Clinical Centre of Neurosurgery and Neurology, Uzhgorod, Ukraine	Site Investigator	Data acquisition

**Dr Jo Janette De la Calzada**	Cebu Doctors' University Hospital, Cebu City, Philippines	Site Investigator	Data acquisition
**Dr Anne de Saint Martin**	Centre Hospitalier Universitaire de Strasbourg-Hôpital Hautepierre, Service de Pediatrie 1, Centre Référent des Épilepsies Rares, Strasbourg, France	Site Investigator	Data acquisition
**Dr Dmytro Delva**	Ivano-Frankivsk Regional Children's Clinical Hospital, Ivano-Frankivsk, Ukraine	Site Investigator	Data acquisition
**Dr Tayard Desudchit**	King Chulalongkorn Memorial Hospital, Department of Pediatrics, Chulalongkorn University, Bangkok, Thailand	Site Investigator	Data acquisition
**Dr Gabriella Di Rosa**	Azienda Ospedaliera Universitaria Policlinico G. Martino, UOC Neuropsichiatria Infantile, Messina, Italy	Site Investigator	Data acquisition
**Assist. Prof. Argirios Dinopoulos**	University General Hospital of Attikon, Third University Pediatric Department, Haidari, Greece	Site Investigator	Data acquisition
**Dr Milda Endziniene**	Hospital of Lithuanian University of Health Sciences Kaunas Clinics, Neurology Clinic, Department of Children's Neurology, Kaunas, Lithuania	Site Investigator	Data acquisition
**Dr Viktor Farkas**	Semmelweis Egyetem, I. Sz. Gyermekgyógyászati Klinika,	Site Investigator	Data acquisition

	Csecsemő - és Gyermekneurológiai Osztály, Budapest, Hungary		
**Dr Jose Ferreira**	Pediatric Epilepsy and Neurology Specialists, Tampa, FL, USA	Site Investigator	Data acquisition
**Dr José Carlos Ferreira**	Centro Hospitalar de Lisboa Ocidental - Hospital S. Francisco Xavier, Gabinete de Neurologia Pediátrica, Lisbon, Portugal	Site Investigator	Data acquisition
**Dr András Fogarasi**	Magyarországi Református Egyház Bethesda Gyermekkórháza, Neurológia, Budapest, Hungary	Site Investigator	Data acquisition
**Dr Cassiano Mateus Forcelini**	Hospital São Vicente de Paulo, Unidad de Pesquisa Clinica HSVP, Passo Fundo, Brazil	Site Investigator	Data acquisition
**Prof. Hadassa Goldberg-Stern**	Schneider Children’s Medical Center of Israel, Institute of Pediatric Neurology - Epilepsy Center, Petah Tikva, Israel	Site Investigator	Data acquisition
**Dr Tiziana Granata**	Istituto Neurologico ‘C. Besta’, Fondazione IRCCS Istituto Neurologico Carlo Besta, U.O. Neuropsichiatria Infantile, Milan, Italy	Site Investigator	Data acquisition
**Dr Ioana Grigore**	Spitalul Clinic de Urgenta pentru Copii Sfanta Maria Iasi, Clinica Neurologie Pediatrica, Iasi, Romania	Site Investigator	Data acquisition
**Dr Christian Paul Guzman Astorga**	Hospital General de Culiacán Dr Bernardo J. Gastelum, Culiacán, Mexico	Site Investigator	Data acquisition

**Dr Jose Antonio Infante Cantu**	Hospital Universitario Dr José Eleuterio González, Monterrey, Mexico	Site Investigator	Data acquisition
**Dr Li Jiang**	Children's Hospital of Chongqing Medical University, Department of Neurology, Chongqing, China	Site Investigator	Data acquisition
**Dr Yuwu Jiang**	Peking University First Hospital, Department of Pediatric Neurology, Beijing, China	Site Investigator	Data acquisition
**Dr Pongkiat Kankirawatana**	Children's Hospital of Alabama, Division of Pediatric Neurology, Birmingham, AL, USA	Site Investigator	Data acquisition
**Prof. Yulia Karakulova**	FSBEI HE "Perm State Medical University named after Academician E.A. Wagner Ministry of Health of the Russian Federation", Perm, Russia	Site Investigator	Data acquisition
**Prof. Olga Khaletskaya**	Limited Liability Company "Nizhmedklinika", Nizhny Novgorod, Russia	Site Investigator	Data acquisition
**Dr Volodymyr Kharytonov**	Territorial Medical Association "Psychiatry", Kiev, Ukraine	Site Investigator	Data acquisition
**Prof. Heung Dong Kim**	Severance Hospital, Yonsei University Health System, Seoul, South Korea	Site Investigator	Data acquisition
**Prof. Ki Joong Kim**	Seoul National University Hospital, Seoul, South Korea	Site Investigator	Data acquisition
**Prof. Marija Knezevic Pogancev**	Institute for Child and Youth Health Care of Vojvodina, Department for Developmental Neurology and Epileptology, Novi Sad, Serbia	Site Investigator	Data acquisition

**Dr Liudmila Kraeva**	Federal State Budgetary Educational Institution of Higher Education “Siberian State Medical University of the Ministry of Health of the Russian Federation”, Tomsk, Russia	Site Investigator	Data acquisition
**Assist. Prof. Ruzica Kravljanac**	Institute for Mother and Child health Care of Serbia Dr Vukan Cupic, Child Neurology Service, Belgrade, Serbia	Site Investigator	Data acquisition
**Dr David Kvernadze**	Institute of Neurology and Neuropsychology, Tbilisi, Georgia	Site Investigator	Data acquisition
**Dr Alla Kyrychenko**	Municipal Institution “Dnipropetrovsk Regional Clinical Children’s Hospital”, Dnipro, Ukraine	Site Investigator	Data acquisition
**Dr Volodymyr Kyrychenko**	Municipal Institution “Vinnytsya Regional Psychoneurological Hospital named after academician O.I. Iuschenko”, Vinnytsya, Ukraine	Site Investigator	Data acquisition
**Dr Wang-Tso Lee**	National Taiwan University Hospital, Taipei, Taiwan	Site Investigator	Data acquisition
**Dr Jianmin Liang**	The First Hospital of Jilin University, Department of Pediatric Neurology, Changchun City, China	Site Investigator	Data acquisition
**Dr Elmer Guillermo López-Meza**	Neurociencias Estudios Clínicos, Culiacán, Mexico	Site Investigator	Data acquisition
**Dr Marissa Lukban**	Manila Doctors Hospital, Clinical Trial Office, Manila, Philippines	Site Investigator	Data acquisition

**Dr Olga Lvova**	Municipal Autonomous Institution “Children's City Clinical Hospital No. 9”, Ekaterinburg, Russia	Site Investigator	Data acquisition
**Assist. Prof. Maša Malenica**	Klinicki Bolnicki Centar “Sestre milosrdnice”, Zagreb, Croatia	Site Investigator	Data acquisition
**Dr Maria Margherita Mancardi**	IRCCS Istituto Giannina Gaslini, Unitá Operativa di Neuropsichiatria Infantile, Genova, Italy	Site Investigator	Data acquisition
**Dr Volodymyr Martyniuk**	Ukrainian Medical Rehabilitation Centre for Children with Organic Disorders of NS, MoH of Ukraine, Kiev, Ukraine	Site Investigator	Data acquisition
**Prof. Gia Melikishvili**	MediClub Georgia, Tbilisi, Georgia	Site Investigator	Data acquisition
**Dr Richard Morse**	Dartmouth-Hitchcock Medical Center, Pediatric Neurology, Lebanon, NH, USA	Site Investigator	Data acquisition
**Dr Sylvia Napuri**	Centre Hospitalier Universitaire de Rennes-Hôpital Sud, Département de Médecine de l’enfant et l’adolescent, Rennes, France	Site Investigator	Data acquisition
**Assoc. Prof. Dimitrije Nikolic**	University Children’s Hospital, Neurology Service, Belgrade, Serbia	Site Investigator	Data acquisition
**Prof. Vilem Novak**	Fakultní nemocnice Ostrava, Oddeleni Detské Neurologie, Ostrava-Poruba, Czech Republic	Site Investigator	Data acquisition

**Dr Liliana Maria Nussbaum**	Spitalul Clinic de Urgenta pentru Copii Louis Turcanu, Centrul de Sanatate Mintala pentru Copii si Adolescenti, Timis, Romania	Site Investigator	Data acquisition
**Dr Claudio Palacios**	Sanatorio Allende, Córdoba, Argentina	Site Investigator	Data acquisition
**Prof. Pavel Pilipenko**	State Budgetary Institution of Healthcare of the Novosibirsk Region “State Novosibirsk Regional Clinical Hospital”, Novosibirsk, Russia	Site Investigator	Data acquisition
**Dr Barbara Prawdzic-Senkowska**	Wojewódzki Specjalistyczny Szpital Dzieciecy im. sw. Ludwika sw Krakowie, Oddział Dzieci Starszych z Pododdziałem Neurologicznym, Reumatologicznym, Rehabilitacyjnym, Kraków, Poland	Site Investigator	Data acquisition
**Prof. Igor Prpic**	Klinicki Bolnicki Centar Rijeka (Clinical Hospital Centre Rijeka), Rijeka, Croatia	Site Investigator	Data acquisition
**Dr Roshan Raja**	Child Neurology Specialists, Henderson, NV, USA	Site Investigator	Data acquisition
**Dr Olga Shestakova**	Limited Liability Company “Multi-field Center of Modern Medicine Euromed”, Omsk, Russia	Site Investigator	Data acquisition
**Dr Maria Strachunskaya**	Regional State Budgetary Institution of Health “Smolensk Regional Clinical Hospital”, Smolensk, Russia	Site Investigator	Data acquisition
**Dr Roberto Alfonso Suástegui Román**	Human Science Research Trials, Mexico City, Mexico	Site Investigator	Data acquisition

**Prof. Nino Tatishvili**	EVEX Hospitals M. Iashvili Children's Central Hospital, Tbilisi, Georgia	Site Investigator	Data acquisition
**Dr Salvador Vázquez Fuentes**	Hospital Universitario Dr José Eleuterio González, Monterrey, Mexico	Site Investigator	Data acquisition
**Prof. Federico Vigevano**	Ospedale Pediatrico Bambino Gesù, Unità Operativa di Neurologia, Rome, Italy	Site Investigator	Data acquisition
**Dr Gabriela Adriana Visa**	Spitalul Clinic de Pediatrie Sibiu, Sectia de Pediatrie II, Compartimentul de Neurologie Pediatrica, Sibiu, Romania	Site Investigator	Data acquisition
**Dr Yi Wang**	Children's Hospital of Fudan University, Department of Pediatric Neurology, Shanghai, China	Site Investigator	Data acquisition
**Dr Elza Márcia Yacubian**	Universidade Federal de São Paulo MD, São Paulo, Brazil	Site Investigator	Data acquisition
**Dr Jianmin Zhong**	Jiangxi Provincial Children's Hospital, Department of Neurology, Nanchang, China	Site Investigator	Data acquisition
